# Association between oral health care behaviors and subjective oral health status among Korean older adults: a national study using community health survey data

**DOI:** 10.3389/fpubh.2026.1862304

**Published:** 2026-07-28

**Authors:** Hee-Jung Hong, Ju-Dong Jang

**Affiliations:** 1 Department of Public Health, Graduate School, Konyang University, Daejeon, Republic of Korea; 2 Department of Public Health, Graduate School of Public Health and Welfare, Konyang University, Daejeon, Republic of Korea

**Keywords:** community health survey, Korea, older adults, oral health care behaviors, oral hygiene practices, ordinal logistic regression, subjective oral health, unmet dental care needs

## Abstract

**Background:**

Oral health is an essential component of overall health in older adults. However, evidence on the association between oral health behaviors and subjective oral health status using large-scale population-based data remains limited.

**Methods:**

This study analyzed data from 210,212 Korean adults aged ≥60 years using the 2022–2023 Community Health Survey. Ordinal logistic regression analysis was conducted, and the proportional odds assumption was evaluated using the Brant test. For variables violating this assumption, a partial proportional odds model (PPOM) was applied.

**Results:**

Several sociodemographic, behavioral, and oral health-related factors were significantly associated with subjective oral health status. Women were more likely to report better oral health than men (OR = 1.261, 95% CI = 1.236–1.287), while individuals aged ≥70 years were less likely than those in their 60s (OR = 0.748, 95% CI = 0.734–0.763). Higher education was strongly associated with better oral health (high school: OR = 1.711, 95% CI = 1.672–1.752; ≥college: OR = 2.235, 95% CI = 2.171–2.301). Among health behaviors, toothbrushing after dinner or before sleep showed a strong positive association (OR = 1.482, 95% CI = 1.431–1.535), and walking ≥5 days per week was also associated with better oral health (OR = 1.307, 95% CI = 1.278–1.336). Lower perceived stress was one of the strongest factors (low: OR = 1.733, 95% CI = 1.687–1.779; moderate: OR = 1.401, 95% CI = 1.367–1.437). In contrast, smoking (OR = 0.621, 95% CI = 0.603–0.639), low income (OR = 0.825, 95% CI = 0.741–0.918), and unmet dental care needs (OR = 0.231, 95% CI = 0.223–0.239) were associated with lower odds of being in a better oral health category. The PPOM results further indicated that the effects of several variables varied across levels of oral health status.

**Conclusion:**

Subjective oral health among Korean older adults is influenced by multiple interrelated factors, including sociodemographic characteristics, health behaviors, and access to dental care. These findings highlight the need for comprehensive and stage-specific public health strategies to promote oral health and reduce disparities in aging populations.

## 1 Introduction

Oral health is a fundamental component of healthy aging and a critical determinant of overall well-being in later life (1). The World Health Organization defines oral health as a state that enables individuals to eat, speak, and interact socially without pain or functional limitation, underscoring its impact on physical, psychological, and social dimensions (1). As population aging accelerates worldwide, maintaining oral health has evolved from simple disease control to a core public health priority for preserving functional independence and dignity in old age (2). Despite its importance, oral health status among older adults remains suboptimal. Studies have consistently reported a high prevalence of periodontal disease and edentulism in aging populations, with many individuals experiencing extensive unmet treatment needs (3). At the global level, edentulism and untreated dental caries remain highly prevalent among middle-aged and older adults, indicating persistent unmet needs for oral care in aging societies (3, 4).

South Korea is undergoing a rapid demographic transition and is expected to become a super-aged society by 2025, with adults aged 65 years and older comprising more than 20% of the total population (5). This shift has intensified the burden of chronic oral conditions—such as periodontal disease, extensive tooth loss, and edentulism—that remain common among Korean older adults (6). These conditions are closely linked to systemic health risks, including metabolic syndrome, cardiovascular disease, and cognitive decline (7, 8). In addition, the emerging concept of oral frailty, defined as a multidimensional decline in oral function, has been identified as a significant predictor of physical frailty, disability, and mortality in later life (9, 10).

Subjective oral health status (SROH) is a comprehensive indicator that reflects clinical status, health literacy, and psychological well-being (11). Among older adults, SROH is strongly associated with health-related quality of life, as oral discomfort and functional limitations can undermine daily activities, nutrition, and social participation (12, 13). Longitudinal evidence further suggests that poor SROH increases the risk of depression, reinforcing a vicious cycle between oral and mental health (14). National data from Korea show that older adults report poorer SROH than younger age groups and that income-related inequalities persist despite the expansion of dental insurance (5, 15, 39). Recent Korean and international studies also report that self-rated poor oral health and chewing discomfort are common among socially and economically vulnerable older adults (3, 16, 17).

Individual oral health behaviors are the primary modifiable determinants of SROH in later life. While toothbrushing frequency is a well-established factor, recent evidence emphasizes the importance of brushing timing, particularly before sleep, in preventing periodontal disease and improving SROH (18, 19). The use of interdental cleaning devices and halitosis management further contribute to better periodontal status, social confidence, and oral health–related quality of life (20, 21). Regular dental attendance is also essential; however, many older adults seek care only when symptoms become severe, and studies from Korea and other countries have documented substantial barriers to preventive dental service use (22–24). Beyond specific behaviors, self-regulation frameworks suggest that oral hygiene practices are shaped by motivational and cognitive processes, highlighting the need for interventions that strengthen self-regulatory capacity as well as technical skills (25).

Despite the recognized importance of SROH, significant disparities persist due to socioeconomic and environmental factors. Structural determinants, such as household income, education, residential area, and social capital limit access to dental care and shape oral health trajectories over the life course (26, 27). These outcomes are deeply influenced by the social determinants of health, where factors such as education, income, and insurance coverage play a decisive role in shaping the oral health trajectory of older adults (26). Older adults living alone or in rural areas often experience greater barriers to dental services and report lower SROH, partly because of social isolation and limited healthcare infrastructure (15, 28). Financial constraints contribute to unmet dental care needs, which are strongly associated with poor SROH and reduced quality of life among Korean older adults (29–32). However, few studies have used large-scale, nationally representative data to disentangle the specific impact of daily oral hygiene habits such as brushing timing on SROH in Korean older adults, and even fewer have applied advanced ordinal models that relax the proportional odds assumption (33, 40). Therefore, this study aimed to examine the association between oral health care behaviors and SROH among Korean older adults using data from the 2022–2023 Community Health Survey. By employing a partial proportional odds model, which allows effect estimates to vary across ordered levels of SROH, we sought to provide more nuanced and policy-relevant evidence to guide targeted oral health promotion strategies in a super-aged society.

## 2 Materials and methods

### 2.1 Study design and data source

This study employed a cross-sectional design using data from the 2022 and 2023 Community Health Survey (CHS) conducted by the Korea Disease Control and Prevention Agency (KDCA). The CHS is a nationally representative survey that collects health-related information through a standardized computer-assisted personal interview (CAPI) conducted by trained interviewers at the community level.

A total of 463,537 individuals participated in the CHS during the study period. After excluding respondents with missing values, 210,212 community-dwelling adults aged 60 years or older were included in the final analysis.

### 2.2 Ethical considerations

This study was approved for exemption from review by the Institutional Review Board (IRB) of Konyang University (KYU IRB 2024–09-002). The CHS dataset is publicly available and de-identified, ensuring that individual participants cannot be identified. All analyses were conducted in accordance with relevant ethical guidelines.

### 2.3 Variables

#### 2.3.1 Outcome variable

The outcome variable was subjective oral health status, measured using a self-reported question assessing overall oral health. Responses were categorized into three levels: “good” (very good/good), “moderate,” and “poor” (poor/very poor). Responses such as “do not know” or refusal were treated as missing values and excluded from the analysis.

#### 2.3.2 Sociodemographic variables

Sociodemographic variables included age (60–69 years, ≥70 years), sex (male, female), household income (<2 million KRW vs. ≥2 million KRW), residence area (urban vs. rural), economic activity (yes/no), education level (≤middle school, high school, ≥college), and marital status (married vs. non-married).

#### 2.3.3 Health behavior variables

Health behavior variables included smoking status (current smoker vs. non-smoker), alcohol consumption (non-drinker, low-frequency, high-frequency), body mass index (BMI; underweight, normal, obese), physical activity (vigorous and moderate activity), walking frequency (0 days, 1–4 days/week, ≥5 days/week), perceived stress (high, moderate, low), depressive symptoms (yes/no), and physician-diagnosed hypertension and diabetes (yes/no).

#### 2.3.4 Oral health-related variables

Oral health-related variables included toothbrushing behavior (after lunch, after dinner or before sleep), chewing difficulty, dental visits within the past year, and unmet dental care needs (unmet, met, no need).

### 2.4 Statistical analysis

All statistical analyses were conducted using R version 4.5.0. Descriptive statistics and chi-square tests were performed to examine differences in subjective oral health status according to participant characteristics. To identify factors associated with subjective oral health status, ordinal logistic regression analysis was conducted using a cumulative logit model via the polr function in the MASS package. The outcome variable was coded ordinally as good = 1, moderate = 2, and poor = 3, such that higher values reflect worse subjective oral health. In Models 1–4 (MASS:polr), an odds ratio (OR) greater than 1 indicates a higher probability of being in a worse oral health category, and OR less than 1 indicates tendency toward better oral health. The proportional odds assumption was evaluated using the Brant test. Because violations of this assumption were observed (*p* < 0.001), a partial proportional odds model (PPOM) was applied using the vglm function in the VGAM package with cumulative (parallel = FALSE) family. Note that the VGAM cumulative function uses the opposite sign convention from polr: in Table 6, OR greater than 1 indicates higher probability of being in a better oral health category, and OR less than 1 indicates lower probability of being in a better category (i.e., more likely to remain in a worse state). Variables violating the proportional odds assumption were modeled with non-parallel coefficients across cumulative thresholds while variables satisfying the assumption retained constrained (proportional) parameters, preserving parsimony and interpretability. This partial approach represents a key methodological contribution, allowing variable-specific effects to be estimated at each threshold rather than assuming a uniform effect across all transitions. Hierarchical models were constructed sequentially: Model 1 included sociodemographic variables, Model 2 added health behaviors, Model 3 included chronic disease variables, and Model 4 incorporated oral health-related variables. The PPOM (Model 5) was applied to variables from Model 4. Model fit was assessed using −2 log likelihood, Akaike Information Criterion (AIC), and Bayesian Information Criterion (BIC). Statistical significance was set at *p* < 0.05, and all tests were two-tailed.

## 3 Results

### 3.1 Distribution of subjective oral health status by sociodemographic characteristics

Chi-square tests indicated that all sociodemographic variables were significantly associated with subjective oral health status (*p* < 0.001). Women reported a slightly lower proportion of “good” oral health than men (18.8% vs. 20.6%), while the proportion reporting “poor” oral health was similar between the two groups. Older adults aged ≥70 years showed a markedly higher proportion of “poor” oral health (51.7%) compared with those aged 60–69 years (35.0%).

Higher income and education levels were associated with more favorable oral health status. Individuals in the higher-income group reported a greater proportion of “good” oral health than those in the lower-income group (23.3% vs. 19.5%). Similarly, participants with higher educational attainment showed a higher proportion of “good” oral health, whereas those with ≤elementary education had the highest proportion of “poor” oral health (54.9%).

Regional differences were observed, with rural residents reporting a higher proportion of “poor” oral health compared with urban residents (45.1% vs. 39.4%). Participants engaged in economic activity showed a higher proportion of “good” oral health (21.6%) and a lower proportion of “poor” oral health compared with those not engaged in economic activity.

Marital status was also associated with oral health status, with married individuals reporting better subjective oral health than unmarried individuals. Detailed distributions are presented in Table 1.

**Table 1:** Distribution of subjective oral health status according to sociodemographic characteristics Unit: *n* (%).

Variables	Category	Total	Subjective oral health status			*χ* ^2^	*p*-value
			Good	Moderate	Poor		
Total		210,212(100)	41,136(19.6)	77,457(36.8)	91,619(43.6)		
Sex	Male	92,850(44.2)	19,093(20.6)	33,383(36.0)	40,374(43.5)	120.461	<0.001
	Female	117,362(55.8)	22,043(18.8)	44,074(37.6)	51,245(43.7)		
Age group	60 ∼ 69 years	101,734(48.4)	23,312(22.9)	42,855(42.1)	35,567(35.0)	5981.529	<0.001
	≥70 years	108,478(51.6)	17,824(16.4)	34,602(31.9)	56,052(51.7)		
Household income^†^	High	1,207(0.6)	281(23.3)	551(45.7)	375(31.1)	77.709	<0.001
	Low	209,005(99.4)	40,855(19.5)	76,906(36.8)	91,244(43.7)		
Residence area^‡^	Urban	54,736(26.0)	11,203(20.5)	21,993(40.2)	21,540(39.4)	557.704	<0.001
	Rural	155,476(74.0)	29,933(19.3)	55,464(35.7)	70,079(45.1)		
Economic activity	Yes	101,511(48.3)	21,921(21.6)	39,639(39.0)	39,951(39.4)	1475.086	<0.001
	No	108,701(51.7)	19,215(17.7)	37,818(34.8)	51,668(47.5)		
Education level	≤Elementary	86,689(41.2)	12,324(14.2)	26,757(30.9)	47,608(54.9)	9488.463	<0.001
	Middle school	42,513(20.2)	8,239(19.4)	16,825(39.6)	17,449(41.0)		
	High school	53,165(25.3)	12,343(23.2)	22,184(41.7)	18,638(35.1)		
	≥College	27,845(13.2)	8,230(29.6)	11,691(42.0)	7,924(28.5)		
Marital status	Unmarried	62,644(29.8)	9,439(15.1)	19,716(31.5)	33,489(53.5)	3619.97	<0.001
	Married	147,568(70.2)	31,697(21.5)	57,741(39.1)	58,130(39.4)		

^†^Household income: <2 million KRW (low) vs. ≥2 million KRW (high).

^‡^Residence area: Urban (metropolitan cities) vs. Rural (non-metropolitan areas).

### 3.2 Distribution of subjective oral health status according to health behavior characteristics

All health behavior variables were significantly associated with subjective oral health status (*p* < 0.001). Smoking was associated with poorer oral health, as current smokers showed a higher proportion of “poor” oral health and a lower proportion of “good” oral health compared with non-smokers.

Differences were also observed according to alcohol consumption. Individuals with low- and high-frequency drinking tended to report more favorable oral health compared with non-drinkers, although the pattern varied across categories. Body mass index (BMI) was associated with oral health status, with underweight individuals showing the highest proportion of “poor” oral health, while those with normal and obese BMI categories showed relatively more favorable distributions.

Physical activity was consistently associated with better oral health. Individuals engaging in vigorous or moderate physical activity had a higher proportion of “good” oral health and a lower proportion of “poor” oral health compared with inactive individuals. Similarly, higher walking frequency was associated with improved oral health status, whereas those who did not walk showed the highest proportion of “poor” oral health.

Psychological factors demonstrated strong associations. Individuals with higher perceived stress and those who experienced depressive symptoms reported substantially higher proportions of “poor” oral health and lower proportions of “good” oral health.

Chronic disease status was also associated with oral health. Participants diagnosed with hypertension or diabetes showed poorer oral health distributions compared with those without these conditions. Detailed distributions are presented in Table 2.

**Table 2:** Distribution of subjective oral health status according to health behavior characteristics Unit: *n* (%).

Variables	Category	Total	Subjective oral health status			*χ* ^2^	*p*-value
			Good	Moderate	Poor		
Smoking status	Non-smoker	186,213(88.6)	37,496(20.1)	70,003(37.6)	78,714(42.3)	1159.803	<0.001
	Smoker	23,999(11.4)	3,640(15.2)	7,454(31.1)	12,905(53.8)		
Alcohol consumption	Non-drinker	117,324(55.8)	20,986(17.9)	40,729(34.7)	55,609(47.4)	1937.856	<0.001
	Low-frequency	62,390(29.7)	13,496(21.6)	25,866(41.5)	23,028(36.9)		
	High-frequency	30,498(14.5)	6,654(21.8)	10,862(35.6)	12,982(42.6)		
Body mass index (BMI)^†^	Underweight	6,215(3.0)	734(11.8)	1,553(25.0)	3,928(63.2)	1348.746	<0.001
	Normal	115,867(55.1)	21,671(18.7)	42,536(36.7)	51,660(44.6)		
	Obese	88,130(41.9)	18,731(21.3)	33,368(37.9)	36,031(40.9)		
Vigorous physical activity	No	182,637(86.9)	34,040(18.6)	66,453(36.4)	82,144(45.0)	1316.419	<0.001
	Yes	27,575(13.1)	7,096(25.7)	11,004(39.9)	9,475(34.4)		
Moderate physical activity	No	149,008(70.9)	27,216(18.3)	53,344(35.8)	68,448(45.9)	1246.592	<0.001
	Yes	61,204(29.1)	13,920(22.7)	24,113(39.4)	23,171(37.9)		
Walking frequency^‡^	None	45,967(21.9)	7,077(15.4)	14,063(30.6)	24,827(54.0)	2742.073	<0.001
	1 ∼ 4 days/week	53,178(25.3)	10,245(19.3)	20,518(38.6)	22,415(42.2)		
	≥5 days/week	111,067(52.8)	23,814(21.4)	42,876(38.6)	44,377(40.0)		
Perceived stress	High	34,684(16.5)	4,908(14.2)	10,065(29.0)	19,711(56.8)	3964.063	<0.001
	Moderate	101,892(48.5)	18,740(18.4)	41,471(40.7)	41,681(40.9)		
	Low	73,636(35.0)	17,488(42.5)	25,921(33.5)	30,227(33.0)		
Depressive symptoms	No	192,710(91.7)	38,960(20.2)	72,439(37.6)	81,311(42.2)	1870.177	<0.001
	Yes	17,502(8.3)	2,176(12.4)	5,018(28.7)	10,308(58.9)		
Hypertension	No	103,273(49.1)	21,569(20.9)	39,654(38.4)	42,050(40.7)	695.015	<0.001
	Yes	106,939(50.9)	19,567(18.3)	37,803(35.4)	49,569(46.4)		
Diabetes	No	163,317(77.7)	33,440(20.5)	61,632(37.7)	68,245(41.8)	1007.706	<0.001
	Yes	46,895(22.3)	7,696(16.4)	15,825(33.7)	23,374(49.8)		

^†^BMI categories: <18.5 kg/m^2^ (underweight), 18.5–22.9 kg/m^2^ (normal), ≥23.0 kg/m^2^ (obese).

^‡^Walking frequency: none, 1–4 days/week, ≥5 days/week.

### 3.3 Distribution of self-rated oral health according to oral health-related behaviors

All oral health-related behavior variables were significantly associated with self-rated oral health (*p* < 0.001). Chewing difficulty showed the strongest association with oral health status. Individuals reporting severe chewing difficulty had an overwhelmingly higher proportion of “poor” oral health, whereas those reporting no discomfort showed the highest proportion of “good” oral health. Toothbrushing behaviors were also associated with oral health status. Individuals who practiced toothbrushing after lunch and after dinner or before sleep showed a higher proportion of “good” oral health compared with those who did not. Unmet dental care needs demonstrated a clear gradient. Individuals with unmet dental care needs showed the highest proportion of “poor” oral health, whereas those without dental care needs reported relatively better oral health status. Detailed distributions are presented in Table 3.

**Table 3:** Distribution of self-rated oral health according to oral health-related behaviors unit: *n* (%).

Variables	Category	Total	Subjective oral health status			χ^2^	*p*-value
			Good	Moderate	Poor		
Chewing difficulty	Very severe	15,123(7.2)	42(0.3)	309(2.0)	14,772(97.7)	93117.28	<0.001
	Severe	47,700(22.7)	1,186(2.5)	8,724(18.3)	37,790(79.2)		
	Fair	35,769(17.0)	1,631(4.6)	17,783(49.7)	16,355(45.7)		
	Mild	64,576(30.7)	14,004(21.7)	33,137(51.3)	17,435(27.0)		
	None	47,042(22.4)	24,273(51.6)	17,504(37.2)	5,265(11.2)		
Toothbrushing after lunch	No	93,754(44.6)	16,173(17.3)	32,597(34.8)	44,984(48.0)	1413.832	<0.001
	Yes	116,458(55.4)	24,963(21.4)	44,860(38.5)	46,635(40.0)		
Toothbrushing after dinner or before sleep	No	15,317(7.3)	1,987(13.0)	3,796(24.8)	9,534(62.2)	2339.821	<0.001
	Yes	194,895(92.7)	39,149(20.1)	73,661(37.8)	82,085(42.1)		
Unmet dental care needs	Unmet	27,975(13.3)	1,900(6.8)	7,279(26.0)	18,796(67.2)	9031.165	<0.001
	Met	153,227(72.9)	30,872(20.1)	59,676(38.9)	62,679(40.9)		
	No need	29,010(13.8)	8,364(28.8)	10,502(36.2)	10,144(35.0)		

### 3.4 Factors associated with self-rated oral health

Ordinal logistic regression analysis was conducted to identify factors associated with self-rated oral health. In Model 1, all sociodemographic variables were significantly associated with oral health status (*p* < 0.05). Older age (≥70 years), low income, rural residence, and economic inactivity were associated with higher odds of poorer oral health. In contrast, higher educational attainment and being married were associated with lower odds of poor oral health.

In Model 2, which additionally included health behavior variables, the overall model fit improved (−2 log likelihood decreased from 427,595.36 to 423,737.57). The associations of sociodemographic variables remained consistent. Among health behaviors, current smoking was strongly associated with poorer oral health. Physical activity, including vigorous and moderate activity as well as walking frequency, was associated with better oral health. Individuals engaging in regular physical activity showed lower odds of poor oral health compared with inactive individuals.

Alcohol consumption showed a modest association, with both low- and high-frequency drinkers demonstrating slightly lower odds of poor oral health compared with non-drinkers. BMI was also associated with oral health status, with underweight individuals showing higher odds of poor oral health. Overall, both sociodemographic characteristics and health behaviors were independently associated with variations in self-rated oral health. Detailed regression results are presented in Table 4.

**Table 4:** Factors associated with self-rated oral health status: ordinal logistic regression analysis (models 1 and 2).

Variables	Category	Model 1			Model 2		
		OR	95% CI for OR		OR	95% CI for OR	
			Low	High		Low	High
Sex	(Ref: male)	1			1		
	Female	0.750	0.737	0.764	0.818	0.802	0.834
Age group	(Ref: 60 ∼ 69 years)	1			1		
	≥70 years	1.285	1.262	1.309	1.276	1.252	1.300
Household income	(Ref: high)	1			1		
	Low	1.203	1.085	1.334	1.210	1.090	1.342
Residence area	(Ref: urban)	1			1		
	Rural	1.104	1.083	1.125	1.054	1.034	1.074
Economic activity	(Ref: yes)	1			1		
	No	1.239	1.218	1.260	1.190	1.169	1.211
Education level	(Ref: ≤elementary)	1			1		
	Middle school	0.673	0.657	0.688	0.698	0.682	0.714
	High school	0.538	0.526	0.551	0.564	0.551	0.577
	≥College	0.388	0.377	0.399	0.425	0.413	0.437
Marital status	(Ref: unmarried)	1			1		
	Married	0.700	0.686	0.714	0.740	0.725	0.754
Smoking status	(Ref: non-smoker)				1		
	Smoker				1.717	1.668	1.766
Alcohol consumption	(Ref: non-drinker)				1		
	Low-frequency				0.873	0.857	0.890
	High-frequency				0.897	0.873	0.921
Body mass index (BMI)	(Ref: underweight)				1		
	Normal				1.544	1.464	1.628
	Obese				0.892	0.878	0.907
Vigorous physical activity	(Ref: no)				1		
	Yes				0.863	0.841	0.885
Moderate physical activity	(Ref: no)				1		
	Yes				0.892	0.875	0.908
Walking frequency	(Ref: none)				1		
	1 ∼ 4 days/week				0.798	0.779	0.818
	≥5 days/week				0.711	0.695	0.726
Model fit	−2 Log Likelihood	427,595.36			423,737.57		
	AIC	427,617.36			423,777.57		
	BIC	427,730.17			423,982.68		

In Model 3, additional adjustment for mental health and chronic disease variables further improved model fit (−2 Log Likelihood = 419,729.24; AIC = 419,781.24; BIC = 419,999.35). Female participants had 21.1% lower odds of reporting poorer oral health compared with males (OR = 0.789, 95% CI = 0.773–0.805). Individuals aged ≥70 years had 30.7% higher odds of poorer oral health compared with those aged 60–69 years (OR = 1.307, 95% CI = 1.283–1.333). Higher odds of poorer oral health were also observed among low-income individuals (OR = 1.215), economically inactive individuals (OR = 1.184), and smokers (OR = 1.657).

Lower stress levels and higher physical activity were associated with reduced odds of poorer oral health, while depression, hypertension, and diabetes were associated with increased odds. In Model 4, oral health behavior variables were added, resulting in further improvement in model fit (−2 Log Likelihood = 411,264.22; AIC = 411,322.22; BIC = 411,619.64). Toothbrushing after lunch (OR = 0.908) and after dinner or before sleep (OR = 0.675) were associated with lower odds of poorer oral health. Unmet dental care needs showed a strong association, with individuals who required but received care (OR = 1.622) and those without perceived need (OR = 4.329) having higher odds of poorer oral health compared with those with unmet needs.

However, the Brant test indicated that the proportional odds assumption was violated, suggesting that the standard ordinal logistic model may not fully capture variable-specific effects across outcome levels (Table 5).

**Table 5:** Extended Model II (Model 3) and conventional ordinal logistic model (Model 4) for factors associated with subjective oral health status.

Variables	Category	Model3			Model4		
		OR	95% CI for OR		OR	95% CI for OR	
			Low	High		Low	High
Sex	(ref: male)	1			1		
	Female	0.789	0.773	0.805	0.793	0.777	0.809
Age group	(ref: 60 ∼ 69 years)	1			1		
	≥70 years	1.307	1.283	1.333	1.336	1.311	1.362
Household income	(ref: high)	1			1		
	Low	1.215	1.095	1.349	1.213	1.092	1.347
Residence area	(ref: urban)	1			1		
	Rural	1.072	1.051	1.092	1.065	1.045	1.086
Economic activity	(ref: yes)	1			1		
	No	1.184	1.163	1.205	1.195	1.174	1.216
Education level	(ref: ≤elementary)	1			1		
	Middle school	0.703	0.687	0.720	0.711	0.695	0.728
	High school	0.573	0.560	0.586	0.584	0.571	0.598
	≥College	0.436	0.424	0.448	0.447	0.435	0.461
Marital status	(ref: unmarried)	1			1		
	Married	0.732	0.718	0.747	0.755	0.740	0.770
Smoking status	(ref: non-smoker)	1			1		
	Smoker	1.657	1.610	1.706	1.611	1.564	1.658
Alcohol consumption	(ref: non-drinker)	1			1		
	Low-frequency	0.885	0.869	0.902	0.885	0.868	0.902
	High-frequency	0.911	0.887	0.936	0.898	0.874	0.922
Body mass index (BMI)	(ref: underweight)	1			1		
	Normal	1.522	1.443	1.606	1.499	1.420	1.583
	Obese	0.873	0.858	0.888	0.863	0.848	0.878
Vigorous physical activity	(ref: no)	1			1		
	Yes	0.860	0.839	0.882	0.857	0.836	0.879
Moderate physical activity	(ref: no)	1			1		
	Yes	0.904	0.887	0.921	0.904	0.887	0.922
Walking frequency	(ref: none)	1			1		
	1 ∼ 4 days/weeks	0.812	0.793	0.833	0.831	0.811	0.852
	≥5 days/weeks	0.736	0.720	0.752	0.765	0.749	0.783
Stress Level	(ref: high)	1			1		
	Moderate	0.675	0.659	0.693	0.714	0.696	0.732
	Low	0.529	0.515	0.543	0.577	0.562	0.593
Depressive symptoms	(ref: no)	1			1		
	Yes	1.357	1.313	1.402	1.251	1.210	1.294
Hypertension	(ref: no)	1			1		
	Yes	1.052	1.034	1.070	1.049	1.031	1.068
Diabetes	(ref: no)	1			1		
	Yes	1.231	1.207	1.257	1.220	1.196	1.246
Toothbrushing after lunch	(ref: no)				1		
	Yes				0.908	0.893	0.924
Toothbrushing after dinner or before sleep	(ref: no)				1		
	Yes				0.675	0.651	0.699
Unmet Dental Care Needs	(ref: yes)				1		
	Needed but received care				1.622	1.583	1.661
	No need				4.329	4.184	4.479

The proportional odds assumption was violated for specific variables (Brant test, *p* < 0.05); PPOM was subsequently applied (see Table 6).

### 3.5 Final model using the partial proportional odds model (PPOM)

In the final model, a partial proportional odds model (PPOM) was applied to relax the proportional odds assumption for variables that violated this assumption in Model 4. The model fit statistics were −2 Log Likelihood = 411,264.22, AIC = 411,322.22, and BIC = 411,619.64, indicating a comparable fit to Model 4. The Brant test identified violations of the proportional odds assumption in the following 14 variables: sex, age group, household income, residential area, education level, marital status, smoking status, alcohol consumption, walking frequency, stress level, depressive symptoms, diabetes, toothbrushing after dinner or before sleep, and unmet dental care needs (all *p* < 0.05). These variables were assigned non-parallel (free) coefficients in the PPOM, while the remaining variables—economic activity, BMI, vigorous physical activity, moderate physical activity, hypertension, and toothbrushing after lunch retained constrained (proportional) parameters. The generalized ordered logit model was also examined as an alternative; however, the PPOM was preferred for greater parsimony. Note that in Table 5 (VGAM), OR > 1 indicates higher probability of being in a better oral health category, and OR < 1 indicates lower probability of being in a better category.

For variables that satisfied the proportional odds assumption (constrained parameters), the effects were consistent across all levels of subjective oral health status. Under the VGAM parameterization, OR > 1 indicates higher probability of being in a better oral health category. Individuals who were not economically active had 16.3% lower odds of being in a better oral health category compared with those who were economically active (OR = 0.837, 95% CI = 0.822–0.852). Participants with normal weight had 33.3% lower odds of being in a better oral health category compared with underweight individuals (OR = 0.667, 95% CI = 0.632–0.704), whereas obese individuals had 15.9% higher odds of being in a better oral health category compared with underweight individuals (OR = 1.159, 95% CI = 1.139–1.179).

Regarding physical activity, individuals engaging in vigorous activity had 16.7% higher odds of being in a better oral health category compared with inactive individuals (OR = 1.167, 95% CI = 1.137–1.197), and those engaging in moderate activity had 10.6% higher odds of being in a better oral health category (OR = 1.106, 95% CI = 1.085–1.127). Toothbrushing after lunch was associated with 10.1% higher odds of being in a better oral health category (OR = 1.101, 95% CI = 1.082–1.120), while hypertension was associated with 4.7% lower odds of being in a better oral health category (OR = 0.953, 95% CI = 0.937–0.970).

For variables in which the proportional odds assumption was relaxed (parallel = FALSE), variable-specific odds ratios were estimated across cumulative thresholds. Under the VGAM parameterization, OR > 1 indicates higher probability of being in a better oral health category. Female participants had 26.1% higher odds of being in a better oral health category compared with males (OR = 1.261, 95% CI = 1.236–1.287). Participants aged ≥70 years had 25.2% lower odds of being in a better oral health category compared with those aged 60–69 years (OR = 0.748, 95% CI = 0.734–0.763).

Low-income individuals had 17.5% lower odds of being in a better oral health category compared with those with middle-to-high income (OR = 0.825, 95% CI = 0.741–0.918), and rural residents had 6.1% lower odds of being in a better oral health category compared with urban residents (OR = 0.939, 95% CI = 0.921–0.957). Higher education levels were associated with progressively higher odds of being in a better oral health category compared with those with elementary education or below, with increases of 40.6, 71.1, and 123.5% for middle school, high school, and college or higher education, respectively.

Current smokers showed significantly lower odds of being in a better oral health category compared with non-smokers (OR = 0.621, 95% CI = 0.603–0.639), indicating that smokers are substantially more likely to remain in poor oral health status rather than progressing toward better oral health categories. Alcohol consumption was associated with higher odds of being in a better oral health category, with low-frequency and high-frequency drinkers showing 13.0 and 11.4% higher odds, respectively, compared with non-drinkers. Walking frequency was also associated with better oral health outcomes, with participants walking 1–4 days per week and ≥5 days per week having 20.3 and 30.7% higher odds, respectively.

Stress levels showed a graded relationship: compared with high stress, moderate and low stress levels were associated with 40.1 and 73.3% higher odds of being in a better oral health category, respectively. Individuals with depressive symptoms had 20.1% lower odds of being in a better oral health category (OR = 0.799, 95% CI = 0.773–0.826), and those diagnosed with diabetes had 18.1% lower odds of being in a better oral health category (OR = 0.819, 95% CI = 0.803–0.836).

Among oral health behaviors, toothbrushing after dinner or before sleep was associated with 48.2% higher odds of being in a better oral health category compared with those who did not practice this behavior (OR = 1.482, 95% CI = 1.431–1.535). Regarding dental care utilization, individuals who needed and received dental care had 38.3% lower odds of being in a better oral health category compared with those with unmet needs (OR = 0.617, 95% CI = 0.602–0.632), while those who reported no need for dental care had 76.9% lower odds (OR = 0.231, 95% CI = 0.223–0.239).

Overall, the PPOM results indicate that the effects of several variables vary across levels of subjective oral health status, suggesting that the conventional proportional odds assumption may oversimplify these relationships. The use of PPOM allows for a more flexible and nuanced understanding of how different factors influence the progression of oral health status among older adults (Table 6).

**Table 6:** Factors associated with subjective oral health status using the partial proportional odds model (PPOM) among Korean older adults.

Variables	Category	Model5		
		OR	95% CI for OR	
			Low	High
Sex	(ref: male)	1		
	Female	1.261	1.236	1.287
Age group	(ref: 60 ∼ 69 years)	1		
	≥70 years	0.748	0.734	0.763
Household Income	(ref: middle–High)	1		
	Low	0.825	0.741	0.918
Residential Area	(ref: urban)	1		
	Rural	0.939	0.921	0.957
Economic Activity	(ref: yes)	1		
	No	0.837	0.822	0.852
Education	(ref: ≤elementary)	1		
	Middle school	1.406	1.373	1.440
	High school	1.711	1.672	1.752
	≥College	2.235	2.171	2.301
Marital Status	(ref: unmarried)	1		
	Married	1.325	1.298	1.351
Smoking	(ref: non-smoker)	1		
	Smoker	0.621	0.603	0.639
Alcohol Consumption	(ref: non-drinker)	1		
	Low frequency	1.130	1.108	1.152
	High frequency	1.114	1.084	1.144
Body mass index (BMI)	(ref: underweight)	1		
	Normal	0.667	0.632	0.704
	Obese	1.159	1.139	1.179
Vigorous Physical Activity	(ref: no)	1		
	Yes	1.167	1.137	1.197
Moderate Physical Activity	(ref: no)	1		
	Yes	1.106	1.085	1.127
Walking frequency	(ref: None)	1		
	1 ∼ 4 days/week	1.203	1.173	1.233
	≥5 days/week	1.307	1.278	1.336
Stress Level	(ref: high)	1		
	Moderate	1.401	1.367	1.437
	Low	1.733	1.687	1.779
Depressive symptoms	(ref: no)	1		
	Yes	0.799	0.773	0.826
Hypertension	(ref: no)	1		
	Yes	0.953	0.937	0.970
Diabetes	(ref: no)	1		
	Yes	0.819	0.803	0.836
Toothbrushing after lunch	(ref: no)	1		
	Yes	1.101	1.082	1.120
Toothbrushing after dinner or before sleep	(ref: no)	1		
	Yes	1.482	1.431	1.535
Unmet Dental Care Needs	(ref: Yes)	1		
	Needed but received care	0.617	0.602	0.632
	No need	0.231	0.223	0.239

PPOM – proportional odds assumption relaxed for specific variables.

## 4 Discussion

This study investigated the determinants of subjective oral health status (SROH) among Korean older adults using nationally representative data from the 2022–2023 Community Health Survey (CHS). By employing a partial proportional odds model (PPOM), this research addressed the limitations of the conventional proportional odds assumption and provided a more nuanced estimation of how sociodemographic, behavioral, and psychological factors influence different thresholds of perceived oral health.

Age emerged as a critical factor. In the PPOM (Table 6, VGAM), individuals aged ≥70 years had 25.2% lower odds of being in a better oral health category compared with those in their 60s (OR = 0.748, 95% CI = 0.734–0.763), indicating that advanced age substantially limits the likelihood of favorable oral health. This pattern likely reflects cumulative physiological deterioration, including progressive tooth loss and reduced masticatory function, which are core components of oral frailty (9). As South Korea transitions into a super-aged society, these findings underscore the role of oral health as a primary indicator of systemic vulnerability and healthy aging (1, 5). Consistent with previous literature, women demonstrated 26.1% higher odds of being in a better oral health category than men (OR = 1.261, 95% CI = 1.236–1.287), which may be attributable to greater health literacy and more frequent engagement in preventive hygiene behaviors among women (6, 34).

Socioeconomic status, particularly education, showed a robust association with SROH. Higher educational attainment likely facilitates better access to oral health information and fosters effective self-care behaviors (26, 35). This relationship can be further understood through the lens of top-down self-regulation processes, where higher health literacy and cognitive control enable individuals to maintain consistent oral hygiene routines despite daily barriers (25). While income level showed some variability in the PPOM, its impact on unmet dental care needs remains a significant structural barrier. Our results indicate that economic constraints are directly linked to poor SROH, aligning with global evidence that financial barriers lead to delayed treatment and exacerbate oral health disparities (31, 32).

The importance of maintaining natural teeth and healthy periodontal status extends beyond oral comfort to systemic health. Our findings on the association between oral hygiene and health perception are particularly relevant given that periodontitis and tooth loss have been linked to broader physiological imbalances, including circadian syndrome and metabolic disturbances in aging populations (36). This systemic connection underscores the necessity of integrated health management for older adults.

A pivotal finding of this study is the significant impact of oral hygiene timing. Toothbrushing after dinner or before sleep was a stronger predictor of favorable SROH than brushing at other times. This is consistent with recent findings by Son (19) and Lim and Nam (18), who demonstrated that bedtime oral care is critical for plaque control and long-term periodontal health. To improve these behaviors on a national scale, Korea could draw lessons from long-standing initiatives like Japan’s “8,020 Campaign,” which has successfully promoted the goal of retaining at least 20 natural teeth through consistent community-based oral health promotion (10, 18, 19). Furthermore, the psychological benefits of maintaining oral hygiene such as reducing halitosis and improving interdental cleanliness likely contribute to greater social confidence and overall life satisfaction among older adults (20, 21).

The present study also confirmed a strong link between mental health and oral health. Lower stress levels and the absence of depressive symptoms were significantly associated with better SROH. This relationship is likely bidirectional: psychological distress can lead to neglect of self-care, while chronic oral discomfort can exacerbate social isolation and depression (14, 37). For older adults, particularly those living alone, social capital and emotional well-being may therefore play a mediating role in maintaining adequate oral hygiene practices (15, 28).

A nuanced interpretation of the PPOM results is essential given the different parameterizations of polr (Models 1–4) and VGAM (Table 6). In Table 6, OR < 1 indicates lower probability of being in a better oral health category. Therefore, current smoking (OR = 0.621) and depressive symptoms (OR = 0.799) reflect significantly reduced likelihood of attaining a better oral health category, indicating that tobacco use and psychological distress act as formidable barriers keeping older adults in poor oral health status (14, 36). It should be noted that some associations may reflect reverse causation or reporting bias inherent to cross-sectional data. Conversely, variables with OR>1 in Table 6 such as bedtime toothbrushing (OR = 1.482), higher education, and lower stress levels indicate significantly higher probability of being in a better oral health category, aligning with established epidemiological evidence (8, 19).

The observed association between low- to moderate-frequency alcohol consumption and better SROH warrants cautious interpretation (Table 6). Alcohol consumption (low-frequency OR = 1.130; high-frequency OR = 1.114) shows higher odds of being in a better oral health category compared with non-drinkers. This pattern has been attributed in prior studies to the “healthy drinker effect,” whereby abstainers may include former drinkers who quit due to existing illness, thereby inflating the apparent benefit among current drinkers (34). Social drinking behaviors may also co-occur with higher socioeconomic status and active social engagement, both independently associated with better SROH. Residual confounding from these unmeasured factors cannot be excluded, and this association should not be interpreted as evidence that alcohol consumption improves oral health. Regarding hypertension (OR = 0.953 in Table 6), the slightly lower odds of being in a better oral health category among hypertensive individuals indicates a minor negative association with SROH. However, this close-to-unity odds ratio suggests that the actual impact is very modest. A plausible explanation for this buffered effect is that individuals with diagnosed hypertension have more frequent healthcare contact, which may increase their overall health awareness and potentially counteract further deterioration of oral hygiene. Future studies should examine medication-specific effects and the mediating role of healthcare utilization in this relationship.

The application of the PPOM represents a major methodological contribution of this study. By relaxing the proportional odds assumption only for the 14 variables identified by the Brant test as violating it, this approach balances statistical flexibility with model parsimony a refinement not commonly employed in Korean oral health research. By allowing variable-specific effects to differ across cumulative thresholds, we identified that certain behaviors, such as toothbrushing timing, may have a more pronounced impact on transitioning individuals from poor to moderate oral health than on the transition from moderate to good oral health. This stage-specific insight is often obscured in conventional ordinal logistic models and provides a more accurate framework for designing targeted interventions for older adults at different stages of oral health deterioration.

Despite these strengths including the use of large-scale, nationally representative data and advanced modeling techniques this study has several limitations. The cross-sectional design precludes definitive causal inferences, and self-reported measures of oral health, behaviors, and psychological status may be subject to recall or social desirability bias. In addition, the absence of clinical indicators, such as the number of remaining teeth, periodontal status, or radiographic findings, limits the ability to relate SROH directly to objective disease burden. Furthermore, despite controlling for a broad range of covariates, residual confounding from unmeasured variables such as oral hygiene product use, dental insurance type, frequency of professional dental cleaning, and social support cannot be excluded. The 2022–2023 CHS does not include data on brushing frequency, toothpaste fluoride content, interdental cleaning device use, or mouthwash use, which limits a comprehensive assessment of oral hygiene behavior. Additionally, readers should note that Models 1–4 (polr) and the PPOM (VGAM, Table 6) use opposite sign conventions for OR interpretation. Future longitudinal studies incorporating both subjective and clinical oral health measures, as well as more detailed behavioral indicators, would further validate and extend our findings (36, 38).

The SROH among older adults is influenced by multiple interrelated factors. The high prevalence of chronic oral conditions among older adults necessitates targeted clinical interventions to mitigate the cumulative impact of disease on functional independence (3). Furthermore, because dental attendance is often problem-oriented rather than preventive, effective behavioral interventions are required to promote regular dental check-ups, which have been shown to significantly enhance self-rated oral health and long-term tooth retention (22).

In conclusion, SROH among Korean older adults is shaped by a complex interplay of individual behaviors, mental health, and socioeconomic determinants. The present findings suggest that public health interventions should not only promote general oral hygiene but also specifically emphasize bedtime brushing and regular preventive dental visits as key behaviors for maintaining oral health in late life (23). To effectively mitigate oral health disparities in an aging society, national and local policies should expand financial coverage for preventive dental services, strengthen community-based oral health programs for older adults, and integrate oral health education into broader healthy aging initiatives.

## 5 Conclusion

This study provides a comprehensive analysis of factors associated with subjective oral health status (SROH) among Korean older adults using large-scale, nationally representative data from the 2022–2023 Community Health Survey. By applying both ordinal logistic regression and a partial proportional odds model (PPOM), we identified multidimensional determinants of SROH and accounted for heterogeneous effects that conventional proportional odds models may overlook.

Our findings demonstrate that SROH is shaped by an intricate combination of sociodemographic characteristics, health behaviors, mental health, and specific oral hygiene practices. In particular, higher educational attainment, lower perceived stress, and consistent oral hygiene especially toothbrushing before sleep emerged as robust predictors of favorable SROH, in line with recent domestic and international research (1, 18, 19). Additionally, the substantial negative impact of unmet dental care needs highlights persistent socioeconomic barriers that hinder healthy aging in South Korea (5, 32).

A key methodological contribution of this study is the use of PPOM to capture how the influence of certain determinants varies across different levels of SROH. This approach underscores that public health interventions should be stage-specific, targeting the particular needs of individuals at different points along the continuum from poor to good oral health and across stages of oral frailty (9, 14).

From a public health perspective, our results emphasize the necessity of integrated strategies that combine individual-level behavior modification with structural reforms to reduce socioeconomic inequalities in oral health (2, 27). To effectively mitigate these disparities, public health policies should not only foster regular preventive dental attendance (22) but also adopt innovative digital health strategies. For instance, the implementation of teledentistry could significantly bridge the gap in dental care access for older adults populations residing in rural or underserved areas, ensuring more equitable oral health outcomes (29).

As South Korea enters a super-aged society, developing targeted, multidimensional oral health policies is imperative. Promoting specific evidence-based hygiene habits, expanding access to preventive dental care, and reducing financial and geographic barriers will be essential steps toward improving SROH, enhancing quality of life, and ensuring the overall well-being of the aging population.

## Data Availability

The datasets analyzed in this study are publicly available from the Korea Disease Control and Prevention Agency (KDCA) Community Health Survey website (https://chs.kdca.go.kr) upon reasonable request following the agency’s data access procedures.
